# MRI whole-lesion texture analysis on ADC maps for the prognostic assessment of ischemic stroke

**DOI:** 10.1186/s12880-022-00845-y

**Published:** 2022-07-01

**Authors:** Yuan Zhang, Yuzhong Zhuang, Yaqiong Ge, Pu-Yeh Wu, Jing Zhao, Hao Wang, Bin Song

**Affiliations:** 1 Department of Radiology, Minhang Hospital, Fudan University, 170 Xinsong Road, Shanghai, 201199 People’s Republic of China; 2 Department of Medicine, GE Healthcare, Shanghai, People’s Republic of China; 3 Department of Medicine, GE Healthcare, Beijing, People’s Republic of China; 4 Department of Neurology, Minhang Hospital, Fudan University, Shanghai, People’s Republic of China

**Keywords:** Magnetic resonance imaging, Apparent diffusion coefficient, Texture analysis, Function outcomes

## Abstract

**Background:**

This study aims is to explore whether it is feasible to use magnetic resonance texture analysis (MRTA) in order to distinguish favorable from unfavorable function outcomes and determine the prognostic factors associated with favorable outcomes of stroke.

**Methods:**

The retrospective study included 103 consecutive patients who confirmed unilateral anterior circulation subacute ischemic stroke by computed tomography angiography between January 2018 and September 2019. Patients were divided into favorable outcome (modified Rankin scale, mRS ≤ 2) and unfavorable outcome (mRS > 2) groups according to mRS scores at day 90. Two radiologists manually segmented the infarction lesions based on diffusion-weighted imaging and transferred the images to corresponding apparent diffusion coefficient (ADC) maps in order to extract texture features. The prediction models including clinical characteristics and texture features were built using multiple logistic regression. A univariate analysis was conducted to assess the performance of the mean ADC value of the infarction lesion. A Delong’s test was used to compare the predictive performance of models through the receiver operating characteristic curve.

**Results:**

The mean ADC performance was moderate [AUC = 0.60, 95% confidence interval (CI) 0.49–0.71]. The texture feature model of the ADC map (tADC), contained seven texture features, and presented good prediction performance (AUC = 0.83, 95%CI 0.75–0.91). The energy obtained after wavelet transform, and the kurtosis and skewness obtained after Laplacian of Gaussian transformation were identified as independent prognostic factors for the favorable stroke outcomes. In addition, the combination of the tADC model and clinical characteristics (hypertension, diabetes mellitus, smoking, and atrial fibrillation) exhibited a subtly better performance (AUC = 0.86, 95%CI 0.79–0.93; *P* > 0.05, Delong’s).

**Conclusion:**

The models based on MRTA on ADC maps are useful to evaluate the clinical function outcomes in patients with unilateral anterior circulation ischemic stroke. Energy obtained after wavelet transform, kurtosis obtained after Laplacian of Gaussian transform, and skewness obtained after Laplacian of Gaussian transform were identified as independent prognostic factors for favorable stroke outcomes.

## Background

Stroke has a high mortality and disability rate worldwide [1]. More than 80% of stroke cases are caused by ischemic injury of brain tissue due to reduced acute blood supply [2]. Intravenous thrombolysis, and endovascular therapy have significantly improved outcomes in defined patients with acute ischemic stroke [3]. However, the conservative treatment of patients with stroke beyond the time window remains controversial [4]. Therefore, an accurate evaluation of the prognosis of patients with subacute ischemic stroke would help determine the optimal treatment strategy; thus, improving the survival rate of patients, and reducing the disability rate [5]. 

Currently, computed tomography (CT) and magnetic resonance imaging (MRI) have become important means of diagnosing and evaluating stroke. Diffusion-weighted imaging (DWI) is important in defining the infarct core (IC) of the stroke. This is accomplished by detecting the restricted motion of water molecules due to cytotoxic edema [2, 6]. Previous studies have focused on correlations of the IC volume with collateral circulation and the prognosis of the infarction [7, 8]. Moreover, some studies have attempted to correlate regional mean ADC values with the prognosis in ischemic stroke; therefore, producing conflicting results and a correlation coefficient of only 0.502 [9–11]. While these studies investigated patients with stroke based on morphological and functional information from MRI images, the microscopic alterations of stroke lesions remain unclear.

Texture analysis (TA) is the extraction of texture characteristics from images using computer-based image processing in order to describe the gray level distribution quantitatively and qualitatively as well as the relationship between the pixels within an image. TA can provide high throughput feature information invisible to the naked eye [12–14]. Previous studies have shown that the TA parameters of the MRI images can be used to predict the histological types and grades of head and neck malignancies [15], and distinguish between brain tumors and healthy tissue areas [16]. 

Sarioglu et al. [17] reported that CT-based TA showed high sensitivity (80%) and specificity (70%) in predicting the clinical outcomes of acute ischemic stroke using a mechanical thrombectomy. Regarding MRI-based TA, many studies have performed TA based on T1-weighted and T2-FLAIR images rather than DWI [18]. Thus far, studies have shown that the size of the infarct volume and the collateral circulation are related to the prognosis of stroke [19]. DWI can not only measure the infracts volume, but also reflects the uniformity of the infarct area [20]. Drier et al. [21] suggested that ADC maps can be used to evaluate the volume and growth of IC. So far, the texture features of the ischemic infarction lesions based on ADC have not been widely investigated.

Therefore, we hypothesized that microscopic alterations of stroke lesions might be captured through TA, and these TA parameters may be used as predictors in the automatic prediction of functional outcomes. We investigated the feasibility of using magnetic resonance texture analysis (MRTA) to differentiate favorable function outcomes from unfavorable ones and determine the prognostic factors associated with favorable outcomes.

## Methods

### Study population

The study aligned with the Declaration of Helsinki (revised in 2013). The Institutional Ethics Committee of Minhang Hospital, Fudan University approved this observational, retrospective study (approval number: 2021-008-01 K), and informed consent was waived.

**Fig. 1:**
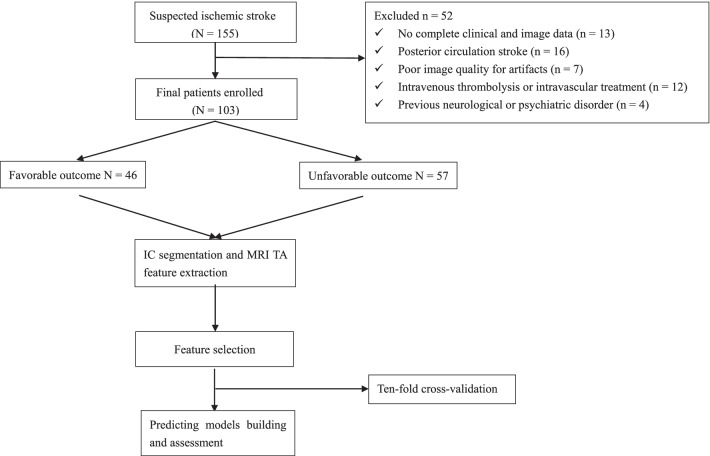
Flow chart of the patient enrollment and the modeling process.* IC* infarcted core,* TA* texture analysis,* MRI* magnetic resonance imaging

From January 2018 to September 2019, 155 patients with confirmed unilateral ischemic stroke who lived independently prior, were consecutively evaluated. In these patients, areas of cytotoxic aedema after symptom onset were defined as hyperintense in DWI with a corresponding hypointensity in the ADC map. The patients were included under the following conditions: (1) age ≥ 18 years; (2) symptom onset time was less than seven days; (3) MRI demonstrated a focal cerebral infarction with neurological deficits; (4) the infarct area was unilateral anterior circulation (three or more consecutive slices of lesions on DWI); (5) complete MRI examination before conservative medical treatment. The patients were excluded under the following conditions: (1) absence of clinical or imaging data; (2) intracranial mass lesion; (3) a history of neurological or psychiatric disorder; (4) posterior circulation stroke; (5) severe MRI artifacts; (6) intravenous thrombolysis or intravascular treatment. Finally, 103 patients (66 males and 37 females, age: 65.43 ± 12.85 years) were enrolled in the study (Fig. 1). Clinical baseline and imaging data were recorded, and contained age, sex, hypertension, blood sugar level, smoking, atrial fibrillation, baseline National Institute of Health Stroke Scale (NIHSS) score on admission (NIHSS_baseline_) and discharge (NIHSS_discharge_), baseline modified Rankin Scale (mRS) score on admission (mRS_baseline_), mRS score on discharge (mRS_discharge_), and a 90-day stroke mRS score. The mRS scores were followed up on day 90, the patients were divided into favorable and unfavorable outcome groups (mRS score ≤ 2 and mRS score > 2, respectively) [4].

### MRI acquisition

A total of 18 patients were imaged using scanner 1 (EXCITE HD 1.5 T MRI; GE Healthcare, Milwaukee, WI, USA) equipped with a head coil (16-channel); MRI examination of 85 patients were performed using scanner 2 (uMR780 3.0 T MRI; United Imaging Healthcare, Shanghai, China) equipped with a head coil (24-channel).

The MRI protocols for scanner 1: (1) for transverse T1-weighted fast spin echo (FSE), with repetition time (TR)/echo time (TE) = 2259/25.4 ms, slice thickness/gap = 5/1.5 mm, bandwidth = 244 Hz/Px, a field of view (FOV) = 240 × 240 mm^2^, and acceleration factor (R) = 2; (2) for transverse T2-weighted FSE, with TR/TE = 5582/111 ms, slice thickness/gap = 5/1.5 mm, bandwidth = 244 Hz/Px, FOV = 240 × 240 mm^2^, and R = 3; (3) for transverse T2-FLAIR, with TR/TE = 8589/88.8 ms, slice thickness/gap = 5/1.5 mm, bandwidth = 244 Hz/Px, and FOV = 240 × 240 mm^2^; (4) for DWI based on single-shot echo planar imaging (SS-EPI), with TR/TE = 3203/83.9 ms, slice thickness/gap = 5/1.5 mm, bandwidth = 3906 Hz/Px, FOV = 240 × 240 mm^2^, R = 2, and b-values of 0 and 1000 s/mm^2^.

The MRI protocols for scanner 2: (1) for transverse T1-weighted FSE, with TR/TE = 2048/11.96 ms, slice thickness/gap = 5/1.5 mm, bandwidth = 180 Hz/Px, FOV = 230 × 200 mm^2^, and R = 2; (2) for transverse T2-weighted FSE, with TR/TE = 4107/88.2 ms, slice thickness/gap = 5/1.5 mm, bandwidth = 180 Hz/Px, FOV = 230 × 200 mm^2^, and R = 2; (3) for transverse T2-FLAIR, with TR/TE = 7500/96.66 ms, slice thickness/gap = 5/1.5 mm, bandwidth = 220 Hz/Px, and FOV = 230 × 190 mm^2^; (4) for DWI-based SS-EPI, with TR/TE = 2800/75.4 ms, slice thickness/gap = 5/1.5 mm, bandwidth = 1790 Hz/Px, FOV = 230 × 220 mm^2^, R = 2, and b-values of 0 and 1000 s/mm^2^.

Meanwhile, ADC maps corresponding to these images were generated automatically using a mono-exponential fitting on workstations from two vendors.

### Lesion segmentation

**Fig. 2:**
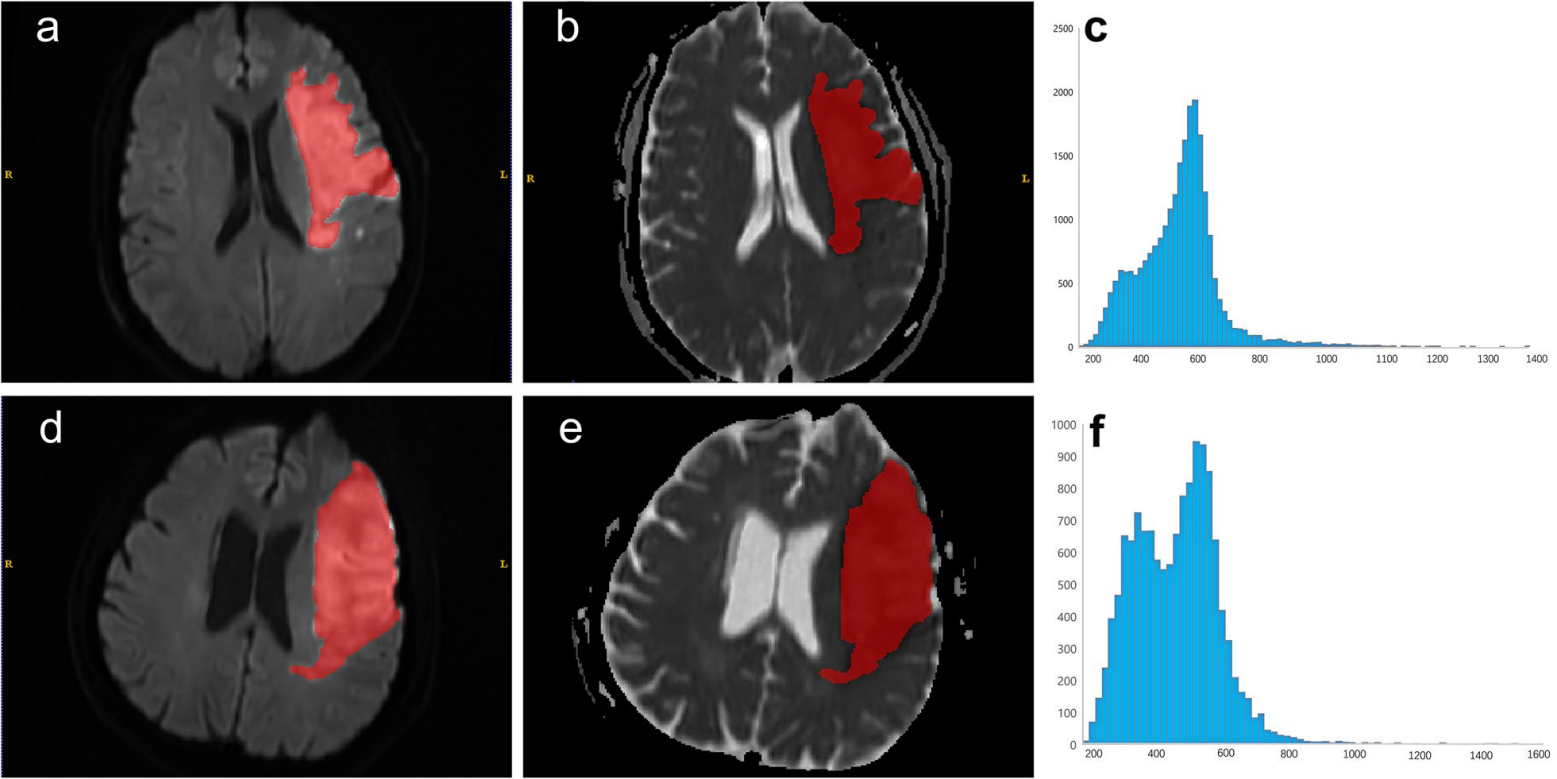
The patient was a 61-year-old male with subacute cerebral infarction in the left basal ganglia and the frontal lobe; the favorable outcome for day 90 mRS score is 2 (**a**–**c**). **a** Axial DWI (b = 1000) shows an obvious uniform hyperintense area (red), the infarct volume was 58.81 ml. **b** The ADC map shows an hypointense area within the diffusion-restricted region (red). **c** ADC histogram, the abscissa is the ADC value, and the ordinate is the pixel value. The patient was a 61-year-old male with a subacute cerebral infarction in the left basal ganglia and the frontal lobe; the unfavorable outcome for the 90-day mRS score is 3 (**d**–**f**). **d** Axial DWI (b = 1000) shows an obvious uniform hyperintense area (red), the infarct volume was 110.71 ml. **e** ADC map shows an hypointense area in the diffusion-restricted region (red). **f.** ADC histogram, the abscissa is the ADC value, and the ordinate is the pixel value

Lesion segmentation was performed after exporting all MRI scans. Two radiologists (with 7 and 15 years of experience in neuro MRIs) first determined the infraction region by consensus using DWI images. The volumes of interest (VOIs) of the largest infraction were manually delineated on the DWI images along the infraction boundaries layer by layer by two radiologists using ITK-SNAP software (package version 3.4.0, www.itksnap.org). In cases involving multifocal infractions, the largest lesion was depicted for further analysis. The VOIs were transferred to the corresponding ADC maps. Figure 2 shows the VOIs within the lesion and the corresponding histograms.

### Texture quantification

Histogram features included 10 quantile, 90 quantile, energy, entropy, interquartile range, kurtosis, skewness, maximum, mean absolute deviation, mean, median, minimum, range, robust mean absolute deviation, root mean squared, total energy, uniformity, and variance in original image type.

Transform image types included wavelet transform and Laplacian of Gaussian transform were extracted through IBSI complied Image Biomarker Standardisation Initiative AK (Analysis Kit Version: 3.2.0; GE Healthcare) software.

A total of 234 features were extracted, intra- and inter- class correlation coefficients (ICCs) were applied to measure the intra- and interobserver agreement for the histogram feature extraction, and features with ICCs > 0.75 indicated a good agreement and were retained for further analysis.

### Texture analysis and model construction

R statistical software v. 3.5.1 (Vienna, Austria) was used for feature selection and model construction. A Mann–Whitney U test was performed for features ICC features > 0.75 in order to explore whether the features were significantly different (*P* < 0.05) between the two groups. Next, univariate logistic regression was used to investigate whether the features were discriminative between the two groups. Then a minimum redundancy maximum relevance (mRMR) was applied to select the most informative features while eliminating redundancy. The retained features were enrolled into a multivariable logistic regression through a backward stepwise selection using a likelihood ratio test to determine the optimal linear combination of the subset of the most predictive features, after which four models were constructed: ADC mode: based on mean ADC value; tADC model: based on texture features on the ADC map (tADC); ADC combined model: ADC mode and clinical characteristics; tADC combined model: tADC mode and clinical characteristics.

In considering the small sample size of the data, 10 times tenfold cross-validation was performed to prove texture analysis was valuable in discriminating one group from another rather than splitting the data into a training and a validation set.

### Statistical analysis

Continuous variables were expressed as the mean ± standard deviation (SD), and discrete variables were expressed in terms of frequency and percentage. To compare the clinical and imaging baseline data of the two groups, SPSS 23.0 software (IBM, Armonk, NY, USA) was used for chi-square or Fisher’s exact testing. The receiver operating characteristic (ROC) curve was evaluated by the following indices, including accuracy, area under the curve (AUC), negative predictive value (NPV), positive predictive value (PPV), sensitivity, and specificity of the prediction model. We used Delong’s test to compare the ROC results of the different models. All statistical analyses were performed with R (version 3.5.1; https: //www.r-project.org) and Python (version 3.5.6; https://www.python.org). A two-tailed *P* value < 0.05 was considered statistically significant.

## Results

### Patient characteristics

A total of 103 patients with unilateral anterior circulation ischemic stroke were enrolled in this study. The demographic, clinical, and imaging characteristics of the patients within the two groups are summarized in Table 1. There were no significant intergroup differences in age, sex, history of hypertension, diabetes, smoking, stroke, scanner, and time from onset to MR. The atrial fibrillation was more common in the unfavorable outcome group, which showed a significantly higher hemorrhage rate and IC volume than the favorable outcome group. The unfavorable outcome group had a significantly lower mean ADC value compared with the favorable outcome group.

**Table 1:** Clinical characteristics of patients in the favorable and unfavorable groups

Variable	Favorable Outcome (N = 46)	Unfavorable Outcome (N = 57)	*P*
Age, years	65.22 ± 12.22	65.60 ± 13.44	0.883
Sex male, n (%)	32 (69.57)	34 (59.65)	0.297
Hypertension, n (%)	28 (60.87)	41 (71.93)	0.235
Diabetes mellitus, n (%)	13 (28.26)	22 (38.60)	0.271
Smoking, n (%)	8 (17.39)	15 (26.32)	0.280
Atrial fibrillation, n (%)	3 (6.52)	14 (24.56)	0.014*
History of stroke, n (%)	6 (13.04)	15 (26.32)	0.096
Homocysteine, mL	16.67 ± 17.03	15.63 ± 13.49	0.503
Hemorrhage, n (%)	3 (6.52)	13 (22.81)	0.023*
IC volume, mL	15.90 ± 16.10	57.05 ± 63.17	0.000*
Mean ADC value (10^−3^ mm^2^/s)	0.40 ± 0.12	0.36 ± 0.08	0.041*
NIHSS_baseline_, median (P_25_, P_75_)	5 (3, 7)	7 (4, 10)	0.000*
NIHSS_discharge_, median (P_25_, P_75_)	4 (1, 7)	7 (5, 10)	0.000*
mRS_baseline_, median (range)	3 (0–4)	4 (1–5)	0.000*
mRS_discharge_, median (range)	2 (0–4)	4 (2–5)	0.000*
Scanner, n (%)			0.306
3.0 T MRI	36 (78.26)	49 (85.96)	
1.5 T MRI	10 (21.74)	8 (14.04)	
Time from onset to MR, hours	57.7 ± 22.0	60.5 ± 18.8	0.485

*IC* infarcted core, *ADC* apparent diffusion coefficient, *NIHSS* National Institutes of Health Stroke Scale, *NIHSS*_*baseline*_ baseline NIHSS score on admission, *NIHSS*_*discharge*_ NIHSS score on discharge, *mRS* modified rankin scale, *mRS*_*baseline*_ baseline mRS score on admission, *mRS*_*discharge*_ mRS score on discharge, *MRI* magnetic resonance imaging

*P < 0.05

### Feature selection and performance

Of all 234 texture features extracted from the ADC maps, average ICCs of the intra- and inter- were greater than 0.75. After performing the Mann–Whitney U test and a univariate logistic regression, we reserved 75 features from the ADC maps for subsequent analysis. Through the mRMR process, we finally retained 30 features from the ADC maps. Backward stepwise multivariable logistic regression selected the most predictive subset features and constructed the final model. Seven texture features were finally retained (Table 2) from transformed images, meaning that the wavelet transform, and the Laplacian of Gaussian transform type of image exhibit better performance, of which Wavelet_LLL_firstorder_energy, Log_sigma_5_mm_ 3D_ firstorder_kurtosis, and Log_sigma_4_mm_3D_firstorder_skewness’s OR value were greater than 1 (*P* < 0.05); thus, these factors were independent risk factors for the prognosis of the patients.

**Table 2:** Multivariate regression of texture features in the tADC and tADC combined model

Model/Texture features	OR	95% CI		Reg coefficient	*P*
*tADC model*					
Wavelet_LLL_firstorder_Energy	16.63	2.22	124.58	2.811	0.006*
Wavelet_LLL_firstorder_Minimum	0.54	0.27	1.08	− 0.617	0.081
Wavelet_LLH_firstorder_Maximum	2.08	0.89	4.86	0.733	0.090
Log_sigma_5_mm_3D_firstorder_Kurtosis	1.76	1.04	3.00	0.568	0.036*
Log_sigma_4_mm_3D_firstorder_Skewness	2.91	1.45	5.84	1.069	0.003*
Log_sigma_2_mm_3D_firstorder_Median	1.66	0.83	3.32	0.509	0.149
Log_sigma_4_mm_3D_firstorder_Maximum	0.49	0.22	1.11	− 0.711	0.088
*tADC Combined model*					
Atrial_fibrillation	1.83	0.99	3.38	0.604	0.054
Wavelet_LLL_firstorder_Energy	8.17	1.53	43.65	2.101	0.014*
Wavelet_LLL_firstorder_Minimum	0.56	0.29	1.10	− 0.572	0.094
Wavelet_LLL_firstorder_Range	2.33	0.87	6.23	0.846	0.092
Wavelet_LLH_firstorder_Maximum	3.16	1.15	8.69	1.150	0.026*
Log_sigma_5_mm_3D_firstorder_Kurtosis	1.47	0.88	2.48	0.388	0.144
Log_sigma_4_mm_3D_firstorder_Skewness	5.01	1.97	12.76	1.611	0.001*
Log_sigma_2_mm_3D_firstorder_Median	2.11	0.96	4.63	0.746	0.063
Log_sigma_4_mm_3D_firstorder_Maximum	0.26	0.09	0.77	− 1.347	0.016*

*tADC* texture features on ADC map, *tADC* Combined, tADC + clinical characteristics, *OR* odds ratio, *CI* confidence interval, *Reg* regression

*P < 0.05

### Model validation

**Fig. 3:**
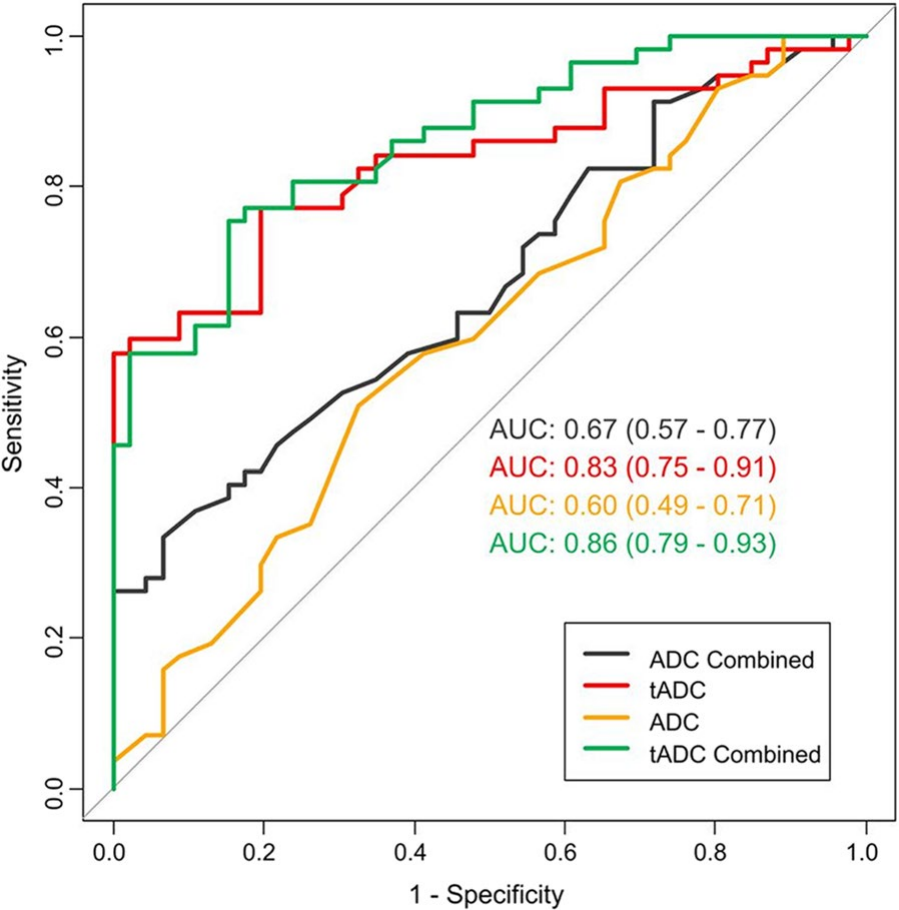
The ROC curve of the models used to assess stroke outcome. *ROC* receiver operating characteristic, *ADC* apparent diffusion coefficient, *tADC* texture features on ADC map

The AUC (95%CI) of the ADC combined, tADC, ADC, and tADC combined models (Fig. 3) were 0.67 (0.57–0.77), 0.83 (0.75–0.91), 0.60 (0.49–0.71), and 0.86 (0.79–0.93), respectively.

**Fig. 4:**
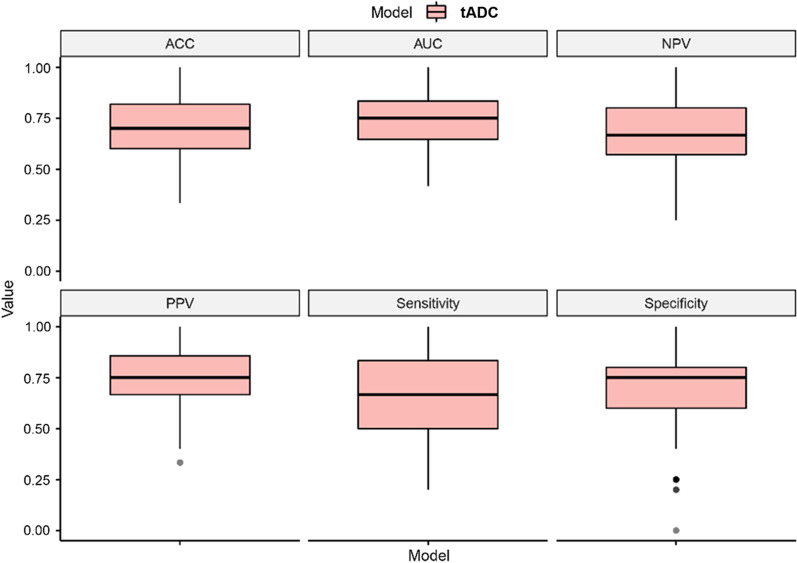
Boxplot of the 100 results of a 10 times tenfold cross-validation

The result of a 10 times tenfold cross-validation is shown in Fig. 4, mean AUC, accuracy (ACC), NPV, PPV, sensitivity, and specificity were 0.75, 0.70, 0.70, 0.71, 0.77, and 0.68, respectively, meaning that the multivariate logistic regression of the tADC model had good stability, and the results were not due to overfitting.

## Discussion

Over the past few years, stroke prognosis has been a research focus and has attracted extensive attention from patients, their families, and clinicians. Furthermore, if therapeutic stroke prevention is not performed rapidly, it often results in a poor prognosis [22]. Moreover, within the treatment window time of 3–6 h, intravenous thrombolysis, which has been proven to be the most vital method to restore cerebral blood flow, can improve the prognosis of the disease [23]. However, there are quite a few patients with stroke beyond that window of time, whose diffusion–perfusion mismatch (DPM) does not meet the requirements for endovascular treatment; thus, the prognosis varies after routine clinical treatment. Therefore, it is of great significance to evaluate the prognosis of the patient beyond the necessary therapeutic time window and prior to treatment.

The patient clinical data have indicated an association between atrial fibrillation and stroke prognosis. The higher incidence of atrial fibrillation in patients revealed unfavorable outcomes and is consistent with the results reported in a previous study [24]. The unfavorable outcome group had a higher rate of hemorrhage compared to that of the favorable outcome group. Hemorrhage transformation is a complex result of multiple factors such as increased vascular permeability and reperfusion injury in the acute stage of ischemic cerebral infarction often leading to unfavorable outcomes [25]. Additionally, previous studies have demonstrated that the clinical function outcomes of patients with acute ischemia can be determined by measuring the IC volume [26, 27]. Our findings corroborated previous studies [28–30] by showing that the IC volume was significantly smaller in the favorable outcome group.

Diffusion-weighted imaging has become a classic MRI sequence technique used for the diagnosis of stroke due to its excellent sensitivity and specificity [2, 31, 32]. As a quantitative parameter of DWI, the ADC value has also been widely applied. Drier et al. [21] reported that ADC values can be performed to evaluate the volume and growth of the infarcts. Brown et al. [33] demonstrated that changes in the ADC values within the IC area are associated with prognosis. Furthermore, a recent study adopted ADC histogram relevant parameters for assessing cerebral small vessel diseases [34]. However, the small number of quantitative features based on the mean ADC value or ADC histogram parameters still limits its application for an accurate prediction of stroke prognosis.

Texture analysis quantifies complex imaging characteristics and has been widely used in various fields, especially in cancer [35–37]. We hypothesized that subtle changes in brain function in patients with stroke could be captured early by measuring neurological alterations related to the texture features within the MRI grayscale images. This study evaluated the predictive value of ADC-based TA for the near-term stroke outcome. Furthermore, we selected features using the mRMR method, which is widely used and can reduce redundancies [38, 39]. 

Furthermore, based on the ADC maps, we can see that the first-order texture features from the wavelet transform, and the Laplacian of Gaussian transform exhibit better performance than the original images. The wavelet transferred images decomposed into a combination of low-frequency images and detail (high-frequency) images, representing different image structures. Therefore, it is easy to extract the structural and detailed information of the original image, while the Laplacian of Gaussian transform used neighboring pixels to improve the contrast of the images; thus, enhancing the details of the images. Wavelet_LLL_firstorder_energy, Log_sigma_5_mm_3D_firstorder_kurtosis, and Log_sigma_4_ mm_3D_firstorder_skewness, showed the best differentiation performance between the favorable and the unfavorable outcome groups. First-order texture features describe the signal distribution within the VOI. Our study only included patients with unilateral anterior circulation infarction; therefore, some patients may have a small DPM or early collateral circulation establishment. These may affect the distribution of the pixel values within the VOI, and thus benefiting the prognosis prediction. For example, a patient had several infarcted areas of similar size. We only selected the largest one, which had less texture information to draw the VOI; however it may have a greater predictive value. The ADC-based TA model has good prognostic performance for stroke (AUC = 0.83, 95%CI 0.75–0.91). Furthermore, the AUC of the combined clinical characteristics model reached a higher AUC of 0.86 (95%CI 0.79–0.93).

Many factors affect the prognosis of stroke. Effective collateral circulation opening and establishment can significantly increase blood perfusion in the infarct area. The larger the ischemic penumbra area, the more ischemic brain tissue can be saved. Therefore, effective collateral circulation and larger penumbra may improve patient outcomes, reduce mortality and the risk of hemorrhage transformation, and patients may benefit more from treatment. In the future, we hope to fully consider these biomarkers and establish a more complete multi-center clinical prediction model.

Admittedly, this study had limitations. First, the statistical results of the single-center retrospective design had a small sample size and need to be verified. Furthermore, the multicenter trials with a larger sample size are necessary. Second, images were acquired using two scanners. Before the texture feature extraction, the images of two scanners were normalized. Third, the use of first-order finite parameters may bias the results. Second-order or even higher-order TA will be used in future studies. Fourth, due to the lack of a validation set, we applied a ten-fold cross-validation approach to compensate, which can avoid over-fitting of the model to a certain extent [40, 41]. Moreover, there was a lack of patients treated with thrombectomy. Further multicentric studies should incorporate patients with this therapeutic modality.

## Conclusion

In conclusion, we established four models based on texture features on ADC maps, mean ADC value, and in combination with patient clinical characteristics. The TA models based on ADC have good accuracy in the prediction of the near-term prognosis of stroke. The diagnostic efficacy of the two models was similar and outperformed the mean ADC models. Wavelet_LLL_firstorder _energy, Log_sigma_5_mm_3D_firstorder_kurtosis, and Log_sigma_4_mm_3D_ firstorder_ skewness were identified as independent prognostic factors for favorable stroke outcomes.

## Data Availability

The datasets generated during and analyzed during the current study are not publicly available due to the huge amount of data (827 Mb), but are available from the corresponding author on reasonable request.

## Abbreviations

CT: Computed tomography
MRI: Magnetic resonance imaging
TA: Texture analysis
MRTA: Magnetic resonance texture analysis
mRS: Modified Rankin scale
ADC: Apparent diffusion coefficient
tADC: Texture features on the ADC map
AUC: Area under the curve
CI: Confidence interval
DWI: Diffusion-weighted imaging
IC: Infarct core
NIHSS: National Institute of Health Stroke Scale
FSE: Fast spin echo
TR: Repetition time
TE: Echo time
FOV: Field of view
SS-EPI: Single-shot echo planar imaging
VOI: Volumes of interest
mRMR: Minimum redundancy maximum relevance
SD: Standard deviation
ROC: Receiver operating characteristic
ICC: Interclass correlation coefficients
PPV: Positive predictive value
NPV: Negative predictive value
DPM: Diffusion–perfusion mismatch
